# Cooling of atoms using an optical frequency comb

**DOI:** 10.1038/s41598-018-38319-3

**Published:** 2019-02-21

**Authors:** N. Šantić, D. Buhin, D. Kovačić, I. Krešić, D. Aumiler, T. Ban

**Affiliations:** Institute of Physics, Zagreb, 10000 Croatia

## Abstract

We report on laser cooling of neutral rubidium atoms by using a single mode of a frequency comb. Cooling is achieved on a dipole-allowed transition at 780 nm in a one-dimensional retro-reflected beam geometry. Temperatures are measured using standard time-of-flight imaging. We show the dependence of the temperature on the cooling time, intensity and detuning of the frequency comb. The lowest temperature achieved is approximately equal to the Doppler temperature and is limited by the intensity of the comb mode driving the cooling transition. Additionally, we verify the analogy between frequency comb and continuous-wave laser cooling. Our work is a step towards laser cooling of atoms with strong cycling transitions in the vacuum ultraviolet, such as hydrogen, deuterium and antihydrogen, where generation of continuous-wave laser light is limited by current laser technology. Achieving efficient cooling at these wavelengths would significantly improve the precision of optical frequency standards, enable measurements of fundamental constants with unprecedented accuracy, improve tests of charge, parity, and time reversal symmetry, and open the way to achieving quantum degeneracy width new atomic species.

## Introduction

Laser cooling and trapping brings atomic physics to one of the most exciting frontiers in science, with applications ranging from atom interferometry^1^ and optical frequency standards^2^ to high precision spectroscopy and ultracold chemistry^3,4^. Regardless of their great importance, laser cooling techniques are still limited to atoms with a simple energy level structure and closed transitions accessible by currently available continuous wave (CW) laser sources.

There are two main obstacles that prevent the extension of laser cooling techniques to a variety of atomic species and molecules. The first is associated with difficulties in creating CW laser light in the vacuum ultraviolet (VUV). The absence of such light sources hampers cooling of simple atoms such as hydrogen, deuterium and antihydrogen that exhibit single-photon transitions suitable for laser cooling in the VUV. For example, cooling of hydrogen and antihydrogen using the strong cycling, single photon 1s-2p transition requires Lyman-alpha radiation at 121 nm, which is far below the limit imposed by the phase matching condition in nonlinear crystals^5^. The second obstacle is associated with the complex level structure of many atoms and all molecules, which permits decay of an excited state into a number of lower lying metastable states, widely separated in energy. Efficient cooling of those species requires multiple repumping lasers which makes the system inefficient and experimentally very challenging^6^.

The aforementioned issues can be addressed using mode-locked femtosecond (fs) or picosecond (ps) lasers with high pulse repetition rates which produce optical frequency combs (FCs). Due to their pulsed light emission, FCs provide high peak powers needed for efficient frequency conversion via nonlinear crystals^7^ or high harmonic generation^8,9^. Simultaneously, FCs preserve long coherence times needed for efficient laser cooling since their spectrum consists of a series of narrow, phase coherent frequency lines. Mode-locked ps lasers have been used to decelerate specific velocity groups in atomic beams that were in resonance with frequency comb modes^10^, and to compress the velocity distribution of an atomic beam^11^. On the other hand, few ps long pulses have been used for broadband laser cooling of ions in the condition when the atomic relaxation time is shorter than the time period of the mode-locked laser^12^. A laser cooling scheme that uses ultrafast pulse trains to cool simple atoms in the VUV as well as complex atoms and molecules is proposed in the work of Kielpinski^13^. The author proposes driving two-photon transition with pulse trains in order to cool atoms with transitions in the VUV. To address cooling of complex atoms and molecules, the author proposes to modulate the original FC at several adjustable RF frequencies via electro-optical modulators. Cooling of atoms on a single-photon transition using a FC has previously been proposed in the work of our group^14^. There, a theory to quantitatively describe the cooling process, based on the interaction of two-level atoms with two counterpropagating pulse trains, is developed and used to derive the radiative force and steady-state temperature. We also showed that simultaneous laser cooling in multiple cooling channels can be achieved using a single frequency comb source.

Along the lines of these proposals, the first experiments that demonstrate FC cooling of atoms and ions have recently appeared in the literature^5,15,16^. Jayich *et al*.^15^ demonstrate FC Doppler cooling of pre-cooled rubidium atoms on the two-photon transition at 778 nm in a 1D counterpropagating geometry. The authors achieved cooling through a coherent process in which multiple excitation pathways are excited by different combinations of comb modes. In the second experiment^5^, the authors demonstrate FC Doppler cooling of trapped magnesium ions on a single-photon transition in the UV by using a frequency tripled comb. And just recently, Ip *et al*.^16^ demonstrated loading, cooling and crystallization of hot ytterbium ions using an optical frequency comb due to phonon lasing of the ion’s harmonic motion in the trap which is driven by the blue-detuned comb teeth.

In this work we demonstrate cooling of rubidium atoms on a dipole-allowed transition at 780 nm by using a FC. To the best of our knowledge, this is the first demonstration of FC cooling of neutral atoms on a single-photon transition. We prepare a cold sample of rubidium atoms that we further cool with the FC in a 1D retro-reflected geometry, achieving a minimum temperature close to the Doppler temperature, *T*_*D*_ = 146 *μ*K^17^. We verify the analogy between FC and CW cooling by replacing the FC with a CW laser in the same experimental geometry, whose intensity matches that of a single comb mode. A simple theoretical model was applied to model our experimental findings, in an approach where one comb line mimics a CW laser interacting with a two-level rubidium atom.

## Results

Our apparatus consists of a standard magneto-optical trap (MOT) for ^87^Rb atoms. A cold rubidium cloud is loaded from background vapor in a stainless steel chamber. The MOT is realized by intersecting six CW laser beams, which, together with the anti-Helmholtz produced quadrupole magnetic field, create the trapping potential. Fluorescence imaging of the cloud is performed with a camera aligned along a horizontal axis. In typical experimental conditions we obtain a cloud 1.6 mm in diameter, which contains ≈10^8^ atoms. By changing the detuning of the MOT beams we are able to prepare a cloud with temperatures in the range of 50–300 μK. Such a cold cloud represents the initial sample for all our measurements presented in this work.

The FC is generated by frequency doubling an Er:fiber mode-locked laser (TOPTICA FFS) operating with a repetition rate *f*_rep_ = 80.5 MHz, an output power of *P* ≈ 230 mW, and a ≈130 nm broad (FWHM) spectrum centered around 1560 nm. The frequency-doubled spectrum used in the experiment is centered around 780 nm with a FWHM of about 5 nm and a total power of 76 mW. Approximately 90000 comb lines are contained under the FC spectral envelope. The FC is stabilized to a CW reference laser (ECDL, Moglabs CEL002) locked to the ^87^Rb|5*S*_1/2_; *F* = 2〉 → |5*P*_3/2_; *F*′ = 3〉 transition, while the pulse train repetition frequency is locked to a stable microwave reference. A brief technical description of the FC stabilization is outlined in Methods.

### FC radiation pressure force

**Figure 1:**
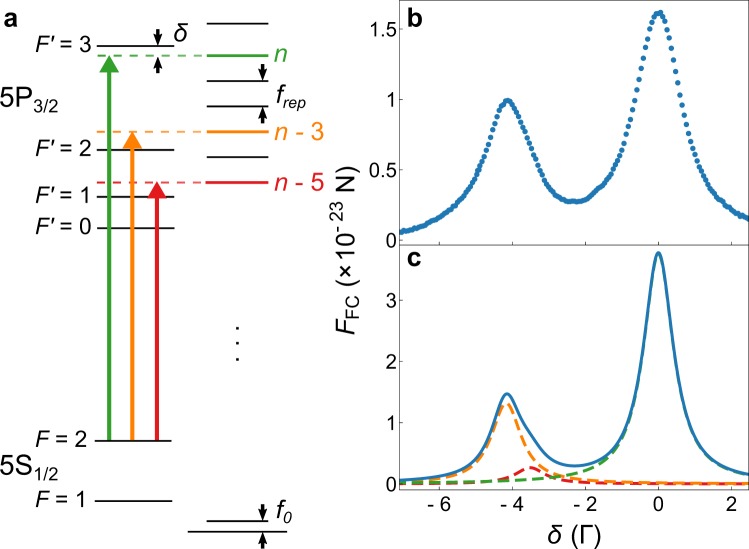
Scheme of relevant ^87^Rb energy levels and FC modes (**a**). Measured (**b**) and calculated (**c**) FC force as a function of detuning. The total force in (**c**), blue line, reflects the interaction with three comb modes: the *n*-th comb mode with the |5*S*_1/2_; *F* = 2〉 → |5*P*_3/2_; *F*′ = 3〉 transition (green dashed line); the (*n* − 3)-rd mode resonance with the |5*S*_1/2_; *F* = 2〉 → |5*P*_3/2_; *F*′ = 2〉 transition (orange dashed line); and the (*n* − 5)-th mode resonance with the |5*S*_1/2_; *F* = 2〉 → |5*P*_3/2_; *F*′ = 1〉 transition (red dashed line).

The efficiency of Doppler cooling depends critically on the scattering rate, since a scattering event changes the atomic momentum, on average, by one photon recoil. Hence, in order to determine the FC scattering rate we measure the FC radiation pressure force on cold Rb atoms. A single linearly polarized FC beam is sent through an acousto-optic modulator (AOM) for fast switching, and is then directed to the center of the MOT where the cold cloud of Rb atoms is prepared. A total power of 18 mW was used with a beam FWHM of 2.7 mm, giving a FC intensity of about 0.01 mW/cm^2^ per comb mode. The measurement sequence starts with the preparation of a cold ^87^Rb cloud and the FC beam off. At *t* = 0 we turn off the MOT beams, and switch on the FC beam. The weak repumper light is left on continuously to optically pump the atoms out of the |5*S*_1/2_; *F* = 1〉 ground state. It propagates perpendicular to the FC, is arranged in a counter-propagating configuration, and has no measurable mechanical effect. We let the FC interact with the cold cloud for 1 ms. During this time the cloud center of mass (CM) accelerates along the FC beam propagation axis (+*x*-direction) due to the FC radiation pressure force. At *t* = 1 ms the FC beam and repumper are switched off, and the cloud expands freely for 5 ms. At *t* = 6 ms the MOT beams are turned on and the cloud fluorescence is imaged with a camera to determine the cloud’s centre of mass displacement in the *x* direction, Δ*x*_CM_. We then use Δ*x*_CM_ to calculate the acceleration of the cloud, which, using the Rb atom mass, finally gives the FC force. This measurement is performed for different relative detunings of the comb lines with respect to the hyperfine transitions, the average of 4 consecutive measurements is shown in Fig. 1(b).

As the FC spectrum consists of identical, regularly spaced comb lines, we characterize the FC spectrum by the detuning *δ* of the *n*-th comb mode from the |5*S*_1/2_; *F* = 2〉 → |5*P*_3/2_; *F*′ = 3〉 transition. Accordingly, the measured FC force dependence on comb detuning, *F*_FC_(*δ*), shown in Fig. 1(b,c) repeats every *f*_rep_ = 80.5 MHz. Two distinct peaks appear in one *f*_rep_ scan reflecting the interaction with three comb modes, as shown schematically in Fig. 1(a). The peak at *δ* = 0 is due to the *n*-th comb mode being in resonance with the |5*S*_1/2_; *F* = 2〉 → |5*P*_3/2_; *F*′ = 3〉 transition, whereas the peak at *δ* ≈ −4.2 Γ is due to the (*n* − 3)-rd mode being in resonance with the |5*S*_1/2_; *F* = 2〉 → |5*P*_3/2_; *F*′ = 2〉 transition, and the (*n* − 5)-th mode in resonance with the |5*S*_1/2_; *F* = 2〉 → |5*P*_3/2_; *F*′ = 1〉 transition. Here, Γ = 2*π* × 6.0666 MHz^17^, and refers to the natural line width of the ^87^Rb |5*S*_1/2_〉 → |5*P*_3/2_〉 transition. The contributions to the FC force coming from transitions to different hyperfine states being in resonance with different comb modes are expressed more clearly in the results of calculations of the FC force shown in Fig. 1(c), where contributions of relevant transitions are explicitly given. The calculated FC force is obtained by numerically solving the optical Bloch equations describing the excitation of six-level ^87^Rb atoms by a FC^18^, and subsequently using the Ehrenfest theorem. More details on the FC force calculation are given in Methods.

The agreement between measured and calculated FC force in Fig. 1(b,c) is reasonable. Relative positions of the two peaks are well reproduced, though there is a small but noticeable broadening of the measured peaks, which we attribute to Zeeman splitting due to the stray magnetic fields. It is worth noting that similar line broadening has also been observed in^15^, whereas a detailed analysis of all possible systematic sources of errors which affect line broadening and shifts in FC spectroscopy can be found in^19^. The comparison of the areas under the measured and calculated force curve gives the factor 1.3, i.e. calculated force is 30% larger than measured. This agreement is satisfactory given that there are no free parameters in the calculations. In addition, calculations do not account for all the physical parameters of the experiment such as the stray magnetic fields, finite laser linewidth, and the laser beam profile.

The largest force is measured when the *n*-th comb mode is resonant with the standard cooling transition for ^87^Rb, i.e., the right peak in Fig. 1(b), for which Δ*x*_CM_ = 0.6 mm is obtained. During 1 ms of FC interaction time the atoms are accelerated with a constant acceleration of 110 m/s^2^ and acquire a velocity of 0.11 m/s. From the measured force, we calculate an on resonance scattering rate of *γ*_scat_ = 18600 s^−1^. Although the measured FC scattering rate is nearly three times larger than the value for a two-photon FC excitation, reported in Jayich *et al*.^15^, both works show comparable performance in term of the peak force, since the momentum transferred per two-photon excitation is about two times the momentum transferred per a single photon excitation. This clearly suggests that FC cooling via one-photon excitation is viable, and could therefore be a good choice when pursuing cooling of atoms and ions with dipole allowed transitions in the VUV spectral region.

### FC cooling

In order to achieve FC cooling in one dimension, we retro-reflect the FC beam. Two counter-propagating FC beams are carefully overlapped in the centre of the MOT, and specific care is taken to slightly focus the retro-reflected beam in order to match its intensity to the incoming FC beam intensity. Prior to entering the MOT chamber *λ*/4 waveplates are put in the beam path, so as to achieve a *σ*^+^*-σ*^−^ polarization configuration.

The measurement sequence for studying FC cooling starts by preparing a cold rubidium cloud in the MOT. At *t* = 0 the MOT beams are switched off. The weak CW repumper laser is again left on. A quadrupole magnetic field with a gradient of 11.3 G/cm remains on during the measurements. Since the cloud is in the centre of the quadrupole field, the magnetic field is *B* ≈ 0. At *t* = 100 *μ*s the FC beams are turned on. The FC beams are on for a time *t*_FC_ (typically 3 ms), which we call the FC cooling time, after which the FC beams are switched off, and the cloud is left to expand freely for several ms before it is imaged with the camera. A series of time-of-flight (TOF) images are taken in this way at different expansion times. We fit our measured atomic distributions to the two dimensional Gaussian function characterized by *σ*_*x*_ and *σ*_*y*_, which are related to the widths of the cloud along the x and y direction, respectively. The obtained spatial width of the cloud *σ*_*x*_ as a function of the expansion time gives an accurate measure of the cloud temperature by fitting to the expression $\sigma {(t)}^{2}={\sigma }_{0}^{2}+\frac{kT}{m}{t}^{2}$^15^, where *t* = 0 is the time when the FC cooling laser is switched off. We repeat the measurement protocol 4 times for a given expansion time, and subsequently average the results to obtain the temperature.

**Figure 2:**
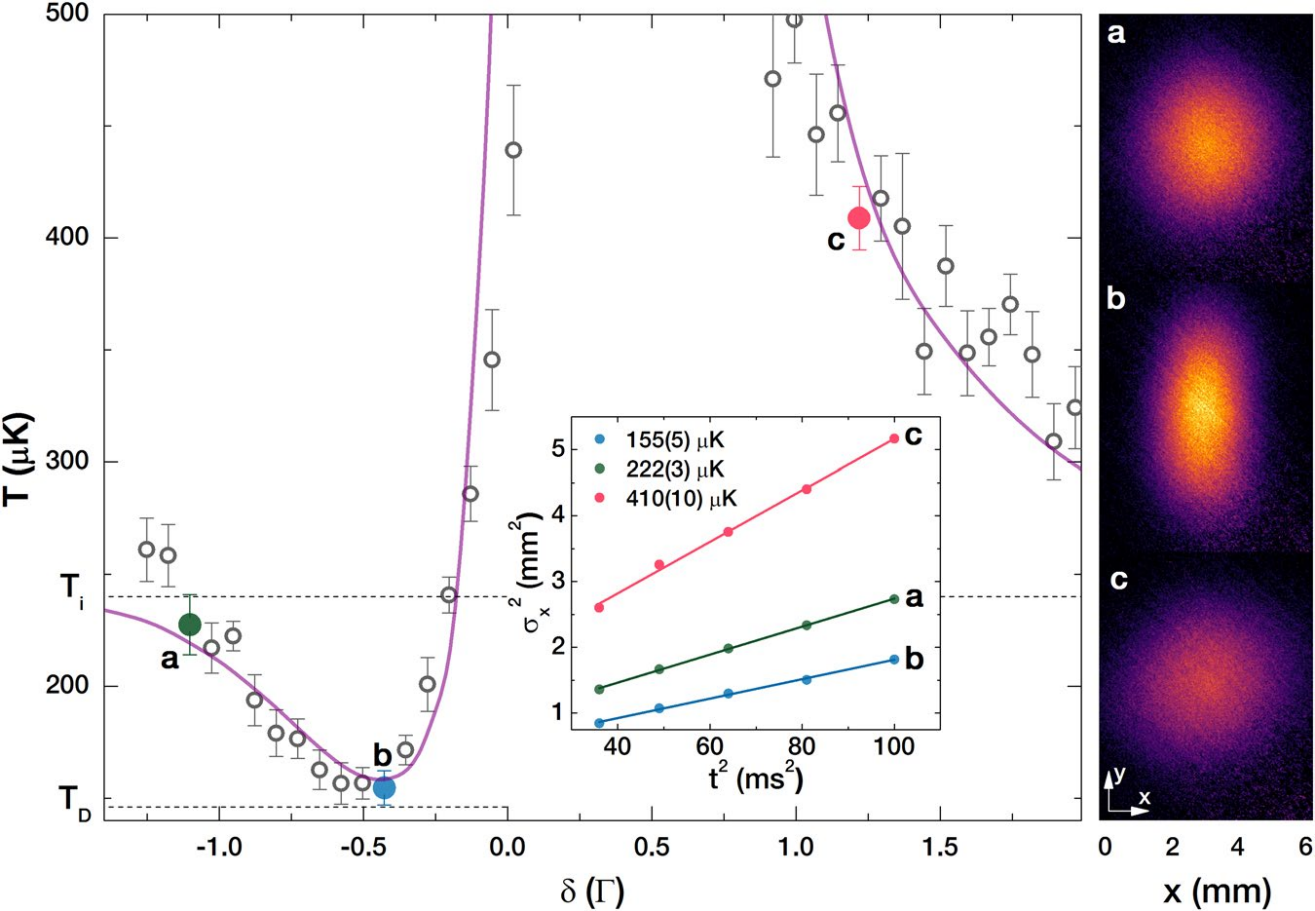
Temperature obtained by TOF after 1D FC cooling as a function of FC detuning. The right panel shows three TOF images taken after 6 ms expansion time for a FC detuning that corresponds to the cloud’s temperature that is close to the initial temperature - green data point (**a**), lowest obtained - blue data point (**b**), higher than the initial temperature - red data point (**c**). Corresponding TOF data for 6–10 ms expansion times are given in the inset. A cloud with an initial temperature of *T*_*i*_ = 240 *μ*K, a FC cooling time of 3 ms, and a comb mode intensity of 0.01 mW/cm^2^ were used. Solid line - theoretical model for relevant experimental parameters.

Temperatures obtained by TOF measurements as a function of *δ* are shown in Fig. 2, where *δ* is the detuning of the *n*-th comb mode from the |5*S*_1/2_; *F* = 2〉 → |5*P*_3/2_; *F*′ = 3〉 transition. A total power of the FC beam entering the MOT chamber was 21 mW and a beam FWHM of 2.8 mm were used, giving 0.6 *μ*W power and 0.011 mW/cm^2^ intensity per comb mode. The initial cloud temperature is *T*_*i*_ = 240 *μ*K, and the FC cooling time is *t*_FC_ = 3 ms.

The measured temperatures approach the initial cloud temperature for large *δ*. FC cooling is observed for the *n*-th comb mode red detuned from the |5*S*_1/2_; *F* = 2〉 → |5*P*_3/2_; *F*′ = 3〉 transition, with the lowest temperature of 155(5) *μ*K is observed for *δ* ≈ −Γ/2. Heating is observed when the *n*-th comb mode is blue detuned from the resonance transition. Figure 2(a) shows the TOF image at 6 ms of expansion time for *δ* = −1.1 Γ, green data point, where the obtained cloud temperature is close to the initial temperature, see green slope in the inset. The measured width (FWHM) of the cloud along the *x*-axis which is relevant for 1D FC cooling is 2.75 mm.

The lowest temperature is observed for *δ* ≈− Γ/2, blue data point, i.e., when there is a comb (*n*-th comb) detuned by ≈−3 MHz from the ^87^Rb |5*S*_1/2_; *F* = 2〉 → |5*P*_3/2_; *F*′ = 3〉 transition. The corresponding TOF image, at 6 ms of expansion time, is shown in Fig. 2(b). As a consequence of the FC cooling the cloud FWHM along the *x*-axis is decreased to 2.17 mm. The temperature given by the slope in the inset is 155(5) *μ*K. This temperature is, within measured uncertainty, in agreement with the predictions for the comb cooling Doppler temperature given in the works of Aumiler *et al*.^14^ and Ip *et al*.^16^ for our experimental parameters *δ* = − Γ/2, *θ* = *π*/160, *T*_*R*_ = 12.5 ns and *ζ* = 1.

For a blue-detuned comb we observe heating, obtaining temperatures higher than the initial temperature, red data point and red slope in the inset. Correspondingly, the TOF image shown in Fig. 2(c) shows an increased cloud FWHM along the *x*-axis of 3.8 mm. The changes in the cloud temperature (along the *x*-axis) with the FC detuning are accompanied with the increase of the temperature in the *y*-axis, i.e., perpendicular to the FC cooling beams, due to the heating caused by spontaneous emission. The signature of this heating along the *y*-axis is evident in the increase of the cloud FWHM along the *y*-axis. Of the ones shown, the largest FWHM along the *y*-axis of 4.33 mm is obtained for the TOF image shown in Fig. 2(b) since the most efficient excitation (and consequently more spontaneous emission) is obtained for this detuning.

**Figure 3:**
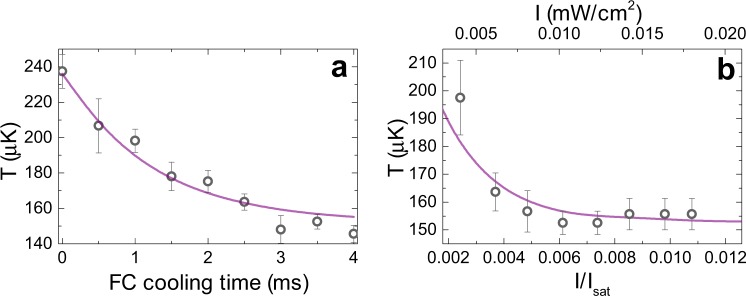
(**a**) The decrease in cloud temperature due to FC cooling as a function of the FC cooling time for an initial cloud temperature *T*_*i*_ = 237 *μ*K, *δ* = −2/3Γ, and single comb mode intensity of 0.01 mW/cm^2^. (**b**) The dependence of the measured temperature on the intensity of the comb mode for a cloud with an initial temperature *T*_*i*_ = 250 *μ*K, a FC cooling time of 3.5 ms and *δ* = −2/3Γ. A theoretical model for relevant experimental parameters is also shown (solid line).

As the FC cooling time is increased, the cloud temperature decreases from the initial value prepared in the MOT phase, *T*_*i*_, and the Doppler limited steady state temperature is achieved after a few ms of FC cooling time. In Fig. 3(a) the dependence of cloud temperature on the FC cooling time is shown, with an initial cloud temperature *T*_*i*_ = 237 *μ*K, *δ* = −2/3 Γ, and single comb mode intensity of 0.01 mW/cm^2^. The steady state is achieved faster for higher comb mode intensities.

The dependence of the measured temperature on the intensity of the comb mode for a cloud with an initial temperature *T*_*i*_ = 250 *μ*K, FC cooling time of 3.5 ms, and for *δ* = −2/3 Γ is shown in Fig. 3(b). Temperatures close to the Doppler temperature are obtained for a wide range of intensities, and an insignificant reduction in temperature is observed when the comb mode intensity approaches zero. Such behavior is a consequence of the very low comb mode intensities which are below 1% of the saturation intensity, *I*_*s*_, for the |5*S*_1/2_; *F* = 2〉 → |5*P*_3/2_; *F*′ = 3〉 transition^17^ and a limited FC interaction time.

## Discussion

The results shown in Fig. 2 imply that the FC cooling is equivalent to the CW laser cooling, since a comb line red-detuned from the cooling transition is needed to obtain cooling. We verify this analogy by performing the same measurements with two counter-propagating CW beams replacing the FC beams, keeping all experimental parameters identical, with the two CW laser beams having intensity equal to the intensity of the relevant comb mode. The obtained temperatures are comparable to the temperatures measured in the case of FC cooling. With respect to this, FC cooling observed in our experiment can be interpreted as single comb mode cooling, i.e., only the *n*-th comb mode is relevant for cooling, while other comb modes do not contribute to cooling or significant heating of the atoms. This is in agreement with the estimation that the scattering rates of neighboring comb modes^13^ are reduced by a factor of (Γ/2*πf*_*rep*_)^2^, which in our experiment gives a factor of 0.0055. This rapid falloff of scattering rate with detuning ensures that, althougth there are many comb lines, the dominant contribution to the total scattering rate comes from the single, near-resonant comb line. In addition, this is in line with the measurement of the FC radiation pressure force, Fig. 1, where it is shown that only one comb mode, the *n*-th comb mode, participates in the excitation of the |5*S*_1/2_; *F* = 2〉 → |5*P*_3/2_; *F*′ = 3〉 cooling transition. This also rules out the concern that the interaction with the |5*S*_1/2_; *F* = 2〉 → |5*P*_3/2_; *F*′ = 1,2〉 resonances, Fig. 1, from which the (n − 3)rd and (n − 5)th comb mode are ≈25 MHz blue detuned, contributes to heating and degrades the minimal observed temperature. In order to model the measured temperatures, we consider the *n*-th comb mode as a CW laser and use the analytical formulas for the calculation of radiation pressure force and diffusion coefficients^20,21^. With these parameters we numerically solve the Fokker-Planck equation for a given FC interaction (cooling) time and extract the atomic velocity distribution after the interaction with the FC, from which the cloud temperature is then obtained. The calculated temperatures, for relevant experimental parameters, are shown in Figs 2 and 3 by solid lines. The agreement between theory and experiment is satisfactory. More details on the temperature calculation are given in Methods.

As seen in Fig. 3(b), our experiment is performed in the low intensity regime ($I\ll {I}_{{\rm{s}}}$) that sets the limit of the observed temperature to the Doppler temperature. In order to achieve sub-Doppler temperatures, i.e., temperatures below 146 *μ*K, the intensity of the comb mode must be increased by a factor of 40. This sub-Doppler threshold was obtained by measuring the temperature as a function of CW laser intensity in the case of CW laser cooling, and it is in agreement with the sub-Doppler threshold given in J. Dalibard and Y. Castin^22^. This leads to the conclusion that sub-Doppler cooling can be achieved with a FC of total output power about 840 mW. Such FC power levels are commercially available, thus the future demonstration of FC sub-Doppler cooling can be anticipated.

## Conclusion

We have demonstrated Doppler cooling of neutral rubidium atoms on a single-photon transition using a single comb line of a frequency comb. The analogy between FC (single comb line) and CW laser cooling is verified by performing the same measurements using a CW laser of appropriate intensity. The minimum temperature obtained is limited by the low intensity in the comb mode relevant for cooling rather than with the residual heating from adjacent comb modes. We believe that in future experiments the power per comb line can easily be increased by more than two orders of magnitude, which should allow cooling to temperatures approaching the recoil limit, thus opening the possibility of laser cooling of species that require light in the VUV spectral region and enabling their use in quantum optics experiments and their applications.

## Methods

### Frequency comb stabilization

The spectrum of the frequency comb (FC) consists of a series of sharp lines, e.g. comb modes^23^. The optical frequency of the *n*-th comb mode is given by *f*_n_ = *nf*_rep_ + *f*_0_, where *f*_rep_ is the laser repetition frequency and *f*_0_ is the offset frequency. Stabilization of the FC requires stabilization of these two degrees of freedom. In our experiment, we actively stabilize *f*_*n*_ and *f*_rep_. We stabilize a FC to a CW reference laser (ECDL, Moglabs CEL002) locked to the ^87^Rb |5*S*_1/2_; *F* = 2〉 → |5*P*_3/2_; *F*′ = 3〉 transition.

The laser repetition frequency, *f*_rep_, is a radio frequency (RF) signal of ≈80.5 MHz. It is detected with a high-speed photodiode and referenced to a rubidium frequency standard referenced low-noise synthesizer. The obtained error signal is used to actively stabilize *f*_rep_ by feedback to the Er:fiber laser intracavity piezoelectric-transducer-mounted mirror.

The optical frequency of the *n*-th comb mode, *f*_n_, is stabilized to an external cavity diode laser (ECDL) which is stabilized to the ^87^Rb |5*S*_1/2_; *F* = 2〉 → |5*P*_3/2_; *F*′ = 3〉 cooling transition using polarization spectroscopy. *f*_n_ is phase locked to the stabilized ECDL by using heterodyne spectroscopy. A fraction of the ECDL light (≈10 mW) and a fraction of the FC light (≈1 mW) are co-propagated and directed first onto a grating to spatially filter the unwanted comb modes to reduce background noise, and then onto a fast photodiode. The measured signal, *f*_beat_, is a radio frequency signal in the range of 0–40 MHz. It is also referenced to a rubidium frequency standard referenced low-noise synthesizer. The obtained error signal is used for stabilization of *f*_n_ via feedback to the Er:fiber laser current.

By changing the reference frequency for the beat note stabilization, it is possible to change the detuning of the *n*-*th* comb mode, *δ*, from the the |5*S*_1/2_; *F* = 2〉 → |5*P*_3/2_; *F*′ = 3〉 transition. The available range for a continuous change of the beat frequency is *f*_*beat*_ = 5–30 MHz, so three scans with different detunings of the reference ECDL are measured and subsequently merged to obtain the total scan of 58 MHz shown in Fig. 1(b), while only one detuning of the reference ECDL is sufficient for the FC scan shown in Fig. 2.

The stability of the FC is evaluated by measuring the optical heterodyne beat note between the FC and an additional ECDL laser stabilized to a two-photon transition in rubidium, which serves as a length primary standard. The length primary standard laser is stabilized to the two-photon transition in ^87^Rb |5*S*_1/2_; *F* = 2〉 → |5*D*_5/2_; *F*′ = 4〉 using a dither lock. The error signal is generated from the laser induced fluorescence (LIF) at 420 nm produced by rubidium atoms contained in a glass cell heated to ≈110 ^o^C, where a photomultiplier tube collects the LIF and generates an adequate signal. The measured Allan deviation which determines the upper limit on the stability of our FC is ≈3 kHz for an integration time of 10 s.

### Theoretical model for the FC radiation pressure force calculation

We model the electric field *E*_*T*_(*t*) of a FC by^24^

1 $${E}_{T}(t)=[\sum _{m=0}^{\infty }\varepsilon (t-m{T}_{R}){e}^{im{{\rm{\Phi }}}_{R}}]{e}^{i{\omega }_{L}t},$$

where *ε*(t) = *E*_0_ *sech* (1.763*t*/*T*_*p*_) is the single pulse envelope, *T*_*p*_ is the pulse length, *T*_*R*_ is the laser repetition period, Φ_*R*_ is the roundtrip phase acquired by the laser within the cavity and the laser spectrum is centered at *ω*_*L*_ + Φ_*R*_/*T*_*R*_. In addition to the FC beam we include a CW beam with amplitude *E*_*cw*_, to model the repumper laser. For the simulation results presented in Fig. 1(c) we have used: *E*_0_ = 5 × 10^4^ V/m, *T*_*p*_ = 300 fs, 1/*T*_*R*_ = 80.54 MHz and Φ_*R*_ = 0 for the FC beam and *E*_*cw*_ = 40 V/m with a detuning of −100 MHz from the |5*S*_1/2_; *F* = 1〉 → |5*P*_3/2_; *F*′ = 2〉 transition for the CW repumper beam. This corresponds to experimental values within measured uncertainty.

Dynamical evolution of the internal atomic states interacting with the FC field *E*_*T*_ and the CW field *E*_*cw*_ is modeled by optical Bloch equations (OBEs). The description of an OBE model for two-level atoms interacting with a FC is given e.g. in Ilinova *et al*.^25^. In our calculations we take into account the full hyperfine level structure of the D_2_(5S_1/2_ → 5P_3/2_) line of ^87^Rb, i.e. a total of 6 levels, see Fig. 1. This results in a system of 21 coupled differential equations for the independent elements *ρ*_*ij*_(*t*) of the density matrix, which are solved numerically. To calculate the steady state values of an optical coherence, we average the solution for a pulse train consisting of 150 pulses over the duration of final 5 pulses. The Doppler shift due to pushing by the FC is neglected due to small velocity acquired (see description of Fig. 1).

We calculate the force *F*_*FC*_ exerted on an atom by a FC pulse train propagating in the +*x*-direction by using the Ehrenfest theorem. The atom-light interaction is approximated to arise solely due to the three near-resonant comb modes. The effective on-resonance Rabi frequency of the *n*-th comb mode is given by ${{\rm{\Omega }}}_{n}=\mu {E}_{n}^{eff}/\hslash$, where $\mu =\sqrt{\mathrm{7/6}}\cdot 3.584\cdot {10}^{-29}$ C · m is the transition dipole matrix element, and ${E}_{n}^{eff}\mathrm{=3.1}$ V/m is the effective electric field amplitude of the *n*-th comb mode. As the powers in the neighboring comb modes are approximately equal, Rabi frequencies of the three modes differ solely due to different coupling strengths of their respective transitions. Ratios of the Rabi frequencies are thus ${\eta }_{n-5}={{\rm{\Omega }}}_{n-5}/{{\rm{\Omega }}}_{n}=\sqrt{\mathrm{1/14}}$ and ${\eta }_{n-3}={{\rm{\Omega }}}_{n-3}/{{\rm{\Omega }}}_{n}=\sqrt{\mathrm{5/14}}$^17^. Coupling of the FC to the *F* = 1 ground state is neglected due to majority of population being in the *F* = 2 ground state, as confirmed by simulations. Following Ilinova *et al*.^25^, the total FC force on a single atom is now given by

2 $${F}_{FC}=\hslash k{{\rm{\Omega }}}_{n}({\eta }_{n-5}\,Im({\rho }_{21^{\prime} })+{\eta }_{n-3}\,Im({\rho }_{22^{\prime} })+Im({\rho }_{23^{\prime} })),$$

where *k* is the laser wavenumber and *ρ*_*ij*′_ is an optical coherence of the transition *F* = *i* → *F*′ = *j*′.

### Theoretical model for a comb mode cooling

In order to model the measured temperatures, we consider the *n*-th comb mode as a CW laser. Temperature is extracted from the width of the atomic velocity distribution obtained after a given interaction time with the laser. Since the atomic velocity distribution after the interaction can still be considered as Maxwell-Boltzman, the temperature is calculated using the relation $m{v}_{rms}^{2}={k}_{B}T$ where *m* is the mass of rubidium atom, *v*_*rms*_ is the standard deviation of the corresponding Gaussian function, *k*_*B*_ is Boltzman constant, and *T* is the temperature.

The atomic velocity distribution after a given interaction time is calculated by employing the Fokker-Planck equation^20^:

3 $$\frac{\partial \rho (v,t)}{\partial t}=-\,\frac{1}{m}\frac{\partial }{\partial v}(F(v)\rho (v,t))+\frac{1}{{m}^{2}}\frac{{\partial }^{2}}{\partial {v}^{2}}(D(v)\rho (v,t)),$$

where *v* is the velocity of the atom, *t* is the interaction time, *ρ*(*v*, *t*) is the atomic velocity distribution, *F*(*v*) and *D*(*v*) are velocity dependent radiation pressure force and diffusion coefficient, respectively.

Radiation pressure force and diffusion coefficients are calculated using standard low-intensity theory for a two-level atom in one dimension^20^.

Total force from two counter-propagating light beams interacting with the ^87^Rb |5*S*_1/2_; *F* = 2〉 → |5*P*_3/2_; *F*′ = 3〉 transition is equal to the sum of the contributions of the two counter-propagating beams:

4 $$F(v)=\frac{\hslash {k}^{2}\delta {{\rm{\Gamma }}{\rm{\Omega }}}^{2}v}{(\frac{{{\rm{\Omega }}}^{2}}{2}+\frac{{{\rm{\Gamma }}}^{2}}{4}+{(\delta -kv)}^{2})(\frac{{{\rm{\Omega }}}^{2}}{2}+\frac{{{\rm{\Gamma }}}^{2}}{4}+{(\delta +kv)}^{2})}\mathrm{.}$$

The Rabi frequency, Ω, is defined by the amplitude of the light electric field, transition dipole matrix element and reduced Planck constant, *k* = 2*π*/780 nm^−1^ is the wave number, and *δ* is detuning from the atomic resonance frequency^17^.

The diffusion coefficient is calculated from:

5 $$D(v)=\frac{{\hslash }^{2}{k}^{2}{{\rm{\Gamma }}{\rm{\Omega }}}^{2}(\frac{{{\rm{\Omega }}}^{2}}{2}+\frac{{{\rm{\Gamma }}}^{2}}{4}+{\delta }^{2}+{(kv)}^{2})}{2(\frac{{{\rm{\Omega }}}^{2}}{2}+\frac{{{\rm{\Gamma }}}^{2}}{4}+{(\delta -kv)}^{2})(\frac{{{\rm{\Omega }}}^{2}}{2}+\frac{{{\rm{\Gamma }}}^{2}}{4}+{(\delta +kv)}^{2})}\mathrm{.}$$

We numerically solve the Fokker-Planck equation for a given set of parameters: atom-light interaction time, *t*_*FC*_, the amplitude of the light electric field, *E*_0_, and detuning, *δ*, and calculate the final atomic velocity distribution from which the temperature of the cloud after the interaction with the light beam is obtained. This temperature corresponds to the temperature measured by TOF spectroscopy, and it is shown by solid lines in Figs 2 and 3. For the simulation results presented in Fig. 2 we used *E*_0_ = 6 V/m and *t*_*FC*_ = 3 ms, while for the simulation results presented in Fig. 3(a,b) *E*_0_ = 8.2 V/m, *δ* = −2/3 Γ and *t*_*FC*_ = 3.5 ms are used, respectively. These results corresponds to the measured values within the measured uncertainty.

## Data Availability

The data that support the findings of this study are available from the corresponding author on reasonable request.
